# To Drive or Not to Drive: Assessment Dilemmas for GPs

**DOI:** 10.1155/2012/417512

**Published:** 2012-01-15

**Authors:** J. Sims, S. Rouse-Watson, P. Schattner, A. Beveridge, K. M. Jones

**Affiliations:** ^1^Healthy Ageing Research Unit, Monash University, Notting Hill, Melbourne, VIC 3168, Australia; ^2^Department of General Practice, Monash University, Notting Hill, Melbourne, VIC 3168, Australia

## Abstract

*Introduction*. Most Australians are dependent on their cars for mobility, thus relinquishing driving licences for medical reasons poses challenges. *Aims*. To investigate how general practitioners (GPs) recognise and manage patients' fitness to drive, GPs' attitudes and beliefs about their role as assessors, and GPs' experiences in assessing and reporting to driving authorities and identify GPs' educational needs. *Methods*. Mixed methods: questionnaire mailed to GPs from three rural and two metropolitan Divisons of General Practice in Victoria, Australia. *Results*. 217/1028 completed questionnaires were returned: 85% recognised a patients' fitness to drive, 54% felt confident in their assessment ability, 21% felt the GP should have primary responsibility for declaring patients' fitness to drive, 79% felt that reporting a patient would negatively impact on the doctor-patient relationship, 74% expressed concern about legal liability, and 74% favoured further education. *Discussion*. This study provides considerable information including recommendations about GP education, the assessment forms, and legal clarification.

## 1. Introduction

Australians are reliant on driving as it allows people to maintain their independence and independence is a key component of positive ageing [1]. Public transport is often inaccessible or unavailable [2], particularly in rural areas. Where and when public transport is available, older people and those with disabilities can experience problems getting on and off public transport or walking to and from stops [1, 2] and can be at greater risk of being killed as pedestrians than are other age groups [2–5].

Regardless of age, cognitive impairment has consistently been shown to increase the risk of crashing [6, 7]. Other factors which affect driving ability include visual defects such as constriction of binocular field, decreased grip, muscle strength and endurance, flexibility and motor speed, decreased neck rotation, arthritis, neurological impairment and falls [6, 8], insulin-dependent diabetes mellitus, and epilepsy [9].

There are increasing numbers of drivers who continue to drive in later life [5, 10, 11]. Given an ageing population and increased morbidity, there will be a greater proportion who have a diminished capacity for driving, with the number of fatalities involving older drivers being estimated to almost triple by 2025 [10]. This prediction is based on a widespread perception that older people are at increased risk of car accidents [11–13]. Calculating the number of crashes per licensed driver and particularly for older drivers is not straightforward, for example, among the “old-old” (≥80 years), many may not drive but keep their licences for identification purposes [9–11, 14]. Overall, older people are not at increased risk of crashing, but they are more susceptible to injury in road traffic accidents [5]. Older people tend to reduce their risk by self-limiting their driving to local trips in daylight hours [5, 15]. Indeed, it has been contended [14] that older people pose much less risk to other road users than do other age groups, yet incur a greater risk of death or serious injury to themselves and their passengers due to their physical vulnerability [1, 16–18].

Regardless of age, relinquishing driving for medical reasons poses personal and professional challenges. With the exception of Western Australia, licensed drivers are legally required to declare any medical conditions or impairments that may affect their ability to drive safely to the relevant driver licensing authority [9]. Victoria is the only state or territory without mandatory medical or on-road tests for all older drivers. Victorian drivers who have been reported to that state's driver licensing authority (VicRoads) must undertake clinical assessments or tests relevant to their impairment, as specified by VicRoads after consideration of clinical advice or other relevant information, such as crash history [19]. Interestingly, older drivers in Victoria perform similarly to drivers in other states in Australia [11]. Evidence suggests that population-based screening is an inappropriate means of assessing the complex and multifactorial basis risk of driving unfitness [5].

Primary care providers (known as general practitioners (GPs)) in Canada and Australia have reported concern about their role in assessing patients' fitness to drive including the risk of socially isolating patients [20–22] and privacy issues [23]. Beyond the national Austroads guidelines [9] which apply to all states and territories and outline health professionals' roles, there are no specific guidelines available for GPs. The Austroads guidelines include information to assist with clinical assessments such as cognitive and/or physical impairment. In addition, all state and territories have a medical report form for the GP or other health professionals to complete and forward to the driver licensing authority, which in Victoria is VicRoads. Drivers can self-report to VicRoads for testing and/or to relinquish their licenses; a medical report can be completed by a GP and forwarded to VicRoads, and VicRoads can provide the driver with information, request that the driver undertakes tests relevant to their impairment, conduct a driving review and/or cancel a driver's licence [9, 19, 24, 25].

The aims of this study were to investigate

1. how GPs recognise and manage patients' fitness to drive, with a particular focus on older patients,
2. GPs' attitudes and beliefs about their role as assessors of a patient's fitness to drive,
3. GPs' experiences in assessing patients and providing reports to driving authorities,
4. identify further educational needs of GPs about driving competence in their patients.

## 2. Method

### 2.1. Design and Instrument Development

A mixed methods design includes semistructured interviews, a focus groups, and a literature search. Three surveys were found during the literature search, providing information for the basis of this questionnaire [20, 26, 27]. Additional questions were developed from qualitative data collected from individual interviews and a focus group. The findings are reported following the four sections of the questionnaire. The qualitative component of the study is documented elsewhere.

### 2.2. The Instrument

The questionnaire included four sections and a total of 68 questions. The questionnaire commenced with an introductory question about frequency of assessing a patient's fitness to drive: if respondents answered “never,” they were asked to complete Section 1 (Demographics) only and return the questionnaire in the reply-paid envelope provided.  Section 2 was about GPs' self-reported practices (triggers for assessing a patient's fitness to drive, frequency of conducting a driving assessment, uncertainty about patients' ability to drive, dealing with the family, and recommending a conditional licence or restrictions); Section 3 was about GPs' attitudes to assessing fitness to drive and contacting the licensing authority, and Section 4 was about educational needs.

### 2.3. Data Collection and Analyses

Participants were purposively sampled to include GPs from both metropolitan and rural Victoria. The questionnaire was mailed to GPs in three rural and two metropolitan divisions of general practice. In Australia, GPs are members of divisions of general practice which are state- and federally funded organisations that provide administrative, technical, and professional development/education support to GPs/practices in their local area. The five Victorian divisions were purposively invited because of the existing positive relationship between the research team and the divisions, and the divisions positive relationship with the GPs/practices in their area. At the time when the project was conducted, there were 29 Divisions in Victoria, of which 11 were rural. Rural divisions were oversampled because public transport appears to be more limited in rural areas, raising greater challenges when assessing driving capacity.

Descriptive statistics were applied. Comparisons between GP subgroups were conducted using Student's *t*-tests and Chi-square tests as appropriate. Data were analysed using statistical package SPSS version 17.

Ethics approval was obtained from the Monash University Human Research Ethics Committee (MUHREC) (CF09/2422-2009001415).

## 3. Results

Of the 1028 questionnaires mailed, 274 were returned, and of those, 14 were “returned to sender” and three were returned “blank.” Of the 257, 40 GPs completed the introductory question about the frequency of assessing a patient's fitness to drive and Section 1 (Demographics) only; 217 completed the full survey (response rate of 21%). Most of these 217 respondents answered all questions; the nonresponse rate to individual items ranged from 1% to 3%. Results in the paper are reported with the percentage in brackets (%).

**Section 1**

The Demographics presented in this section include the 40 GPs who completed the introductory question and Section 1 only, as well as the 217 who completed the full survey. This cohort's characteristics are similar to the national median: the proportion of males (national = 55%) and females (national = 44%), average age (national = 55 years), and practice size (national with >5 = 41%) [28] (Table 1).

**Section 2 (GP Self-Reported Practices)**

Triggers for assessing fitness to drive were reported by respondents as being prompted by being asked to complete a VicRoads medical report form (90%), when the patient was assessed as cognitively impaired (69%), or physically impaired (52%), or diagnosed with a psychiatric illness (25%), when the issue was raised by the patient (75%), by the family or other members of the community (80%), or during a health check (60%). Visual acuity (93%), medication review (78%), cognitive tests (56%), taking a thorough history (56%), and hearing tests (52%) were the most common forms of assessment. Less frequently used were motor function tests (43%) or taking a history from a spouse or other family member (37%). When uncertain about assessment, GPs were most likely to refer to the Austroads handbook (60%) [9] or refer the patient to an occupational therapist (59%) [24]. GPs were less likely to reassess patients periodically to clarify fitness (35%), refer a patient to a medical specialist (26%), or refer to patient reeducation programs such as those available through the Royal Auto Club of Victoria Senior Drive School program (7%) [29]. Half (50%) the GPs usually advised patients with early dementia to stop driving, similarly (46%) usually advised patients with type 2 diabetes to notify the licensing authority of their condition [9]. The GPs were asked how they addressed family and friends' concerns about a patient's fitness to drive. Respondents were most likely (always/frequently) to advise the family or friend to speak to the patient about their concerns (82%), or with the family members' permission the GP would speak to the patient themselves (74%). GPs were less likely to advise the family to contact the driving licence authority (47%), contact the patient and ask the patient to come in to discuss the matter (3%), or advise VicRoads directly (8%). Conditional licensing was viewed as a safe alternative to driving with an unrestricted licence (71%). However, respondents were less likely (25%) to recommend issuing a conditional licence where there were residual concerns about a patient's medical fitness. Respondents were more likely to recommend patients to “not” drive at twilight/night (66%) to drive within a defined kilometre radius from home (62%), avoid driving at busy times (54%), or to drive on minor roads (39%). Few viewed driving with extra mirrors fitted to the car (9%) as a safe alternative.

**Section 3 (GPs' Attitudes to Assessing Fitness to Drive)**

GPs were invited to respond to 14 questions about their attitudes towards assessing driving fitness. Of importance to note is that the majority (85%) felt it was the patient's responsibility to report medical conditions to VicRoads, that there were implications for vehicle insurance if a patient is unfit to drive (93%), that they (the GPs) knew of alternative transport options in their local area (79%), and that reporting a patient to the driving licence authority impacted on the doctor-patient relationship (79%) and led to negative consequences for the patient (79%) (Table 2).

Less than half (46%) the GPs reported having been unduly pressured by patients to reconsider a decision to report the patient to the licensing authority, and only a quarter (23%) experienced patients leaving the practice over licence revocation. Although there was agreement that a GP should be the initial person who assesses fitness to drive (62%), few felt that GPs should be primarily responsible for making this decision (21%), otherwise, the responsibility rests with OTs (54%).

Regarding contacting the drivers licence authority, around a third of the cohort (38%) contacted VicRoads for information about medical guidelines; similarly, few (29%) contacted VicRoads about administrative procedures.

**Section 4 (GP Education)**

The majority indicated that they wanted further education on how to assess fitness-to-drive (74%), functional assessments (70%) and legal obligations (71%). There was mixed response to the format of the education, with more (62%) preferring face-to-face learning (62%) than an online activity (47%). Alerts on medical software (60%), computerised templates (76%), and a one-page quick reference (76%) in conjunction with “Assessing Fitness to Drive” guidelines [9] were tools that could assist GPs to assess patients' fitness to drive.

## 4. Discussion

GPs responses indicated concerns about their role in assessing patients' fitness to drive. Whilst the GPs generally thought it was their responsibility to conduct initial driving assessments, many felt that the final decision should rest elsewhere and that “borderline” cases should be referred to specialist health professionals. While occupational therapists [24] and specially trained medical practitioners may also be involved in assessing driving capacity, patients tend to present to their GP in the first instance. In some regions of Victoria, particularly rural regions, access to other health professionals can be limited. Most respondents were concerned about their legal liability, and the consensus was that VicRoads should take legal responsibility for the final ruling. This is in line with the legislative requirement, but the finding suggests that this cohort was fully aware of this.

GPs did not always recognise when a patient is not fit to drive or should have a driving assessment; rather, the process tended to be reactive, in response to a variety of prompts. When asked what usually triggered the conduct of a driving assessment, most responded when they were asked to complete the VicRoads medical report form. There is scope for GPs to routinely assess patients' cognitive capacity, psychiatric or physical conditions in the health check. Some tests recommended in the literature, such as motor function tests, were virtually unknown to the participating GPs, but tests required by the VicRoads process such as visual acuity tests were mentioned by the GPs [9]. The GPs were unlikely to refer patients to driver re-education program available through the Royal Automobile Club of Victoria [29] because the majority were unaware of the programs or what the program offered. There was also uncertainty about conditional licenses. Most GPs thought that this option was useful when patients were not really fit to drive without restrictions, but recommendations for conditional licences were not made very often.

The GPs recognised the gaps in their competency; less than half were not confident in their abilities. The GPs were asked about education formats and accessing resources and information. Most respondents were open to further education on the “when,” “what,” and “how”s' of driving assessment. Although recognised as an available resource, there had been only limited contact with VicRoads for guidance or advice. Most GPs wanted further education or information about functional assessments and legal obligations [23]. Beyond the Austroads guidelines [9] which refer to the health professionals' role, there are no specific guidelines available for GPs. On the provision of prompting alerts on medical software, a computerised assessment template and a one-page “quick reference” resource based on the Austroads guidelines [9] were all potential options to assist management.

Relinquishing driving has psychological and physical impacts, including increased loneliness, social isolation and mortality risk, dependency on others for transport, and decreased health and participation in out-of-home activities [1, 2, 27]. Although GPs are used to difficult consultations, several felt compromised between being a patient advocate and the need to promote community safety. Privacy was also a concern [23], for example, when approached by the family to deal with the patient, careful judgement is required by the GP. A major difficulty in advising people to stop driving is the major impact on patients' lives, particularly since public transport is not always a realistic alternative in many areas of metropolitan and rural Victoria and indeed in many parts of Australia. Of particular concern was the effect that driving assessments can have on the doctor-patient relationship; many agreed that reporting a patient to the authority could have a negative impact on the relationship. Almost half the GPs felt that they had been unduly pressured by their patients to reconsider their decisions, although only a minority reported having patients leave the practice over revoking a patient's licence.

The response rate (21%) in this study may limit the findings' generalisability, although this response rate is consistent with other surveys in general practice [26]. Given the differing legislative requirements around mandatory medical or on-road tests for all older drivers in the states and territories of Australia, these findings apply solely to Victorian GPs, although the concerns may be of interest and value to GPs in other parts of Australia and other countries such as Canada, where medical practitioners grapple with similar challenges.

## 5. Conclusion and Recommendations

GPs tend to be “reactive” rather than regard driving assessments as a routine part of preventive medicine. Only a quarter of GPs frequently make recommendations for conditional licences, even though most believe that these are safe alternatives for those who are not fit to drive with unrestricted licences. While it is a patient's responsibility to report to VicRoads, it would seem that GPs could be more proactive in this area, given that patients are less likely to know that this is what they should do.

GPs are uncertain about which assessment tools can or should be used within a consultation to help gauge fitness to drive. Beyond the national Austroads guidelines [9] which refer to the health professionals' role, there are no specific guidelines, including guidelines about assessment tools, available for GPs. Not surprisingly, almost threequarters of the cohort indicated a strong interest in further education about driving assessments. Further education may create greater recognition that assessing patients “at risk” of suboptimal driving skills for medical reasons should be a routine preventive measure in general practice.

The national guidelines [9] could be clearer about what to do in “borderline” cases. This is particularly the case for patients with early dementia, but might also apply to people with medical conditions such as diabetes, where the risks of driving are not necessarily well understood by the medical profession. Clarity about legal responsibility for reporting to the driving licensing authority, and publicising this, is required, as is the extent of medicolegal responsibility that rests with GPs needs further clarification.

The doctor-patient relationship can be compromised by having a GP as the final adjudicator on a patient's fitness to drive GPs needs further support in deciding on the driving fitness of borderline cases. This includes clear guidance on when referral is appropriate to OTs and medical specialists. In doubtful cases, GPs should have access to specialised health professionals who can take responsibility for deciding whether a patient is fit to drive.

Based on the findings, the following recommendations are made.

(1) Education programs be developed by relevant organisations such as the Royal Australian College of General Practitioners (RACGP) (which provides access to continuing professional development for GPs), divisions of general practice, universities, and private organisations, on assessing fitness to drive and address (a) indications for conducting assessments, (b) how to conduct and complete an assessment, (c) how to manage the “at risk” group of patients, (d) medicolegal responsibilities, and (e) the availability of community resources.

(2) VicRoads publicise and help clarify the role of the general practitioner, other health professionals, and patients in undertaking driving assessments.

(3) VicRoads assist in clarifying the medicolegal responsibilities of the GP in their role as assessors of fitness to drive.

(4) VicRoads, the state government, insurance companies, and others help inform the public about their responsibilities of informing the appropriate authorities about medical conditions which could affect an individuals' fitness to drive.

## Figures and Tables

**Table 1:** GPs' Demographic characteristics (*n* = 257).

What is your gender?	*n* = 257	Percent
Male	137	58%
Female	101	42%
No response	19	

What is your age?	*n* = 257	Percent

<40 yrs	56	23%
41–60 yrs	153	64%
>60 yrs	30	13%
No response	18	

How many years have you been in practice?	*n* = 257	Percent

<10 yrs	54	23%
10–30 yrs	132	57%
>30 yrs	46	20%
No response	25	

What is the size of the practice in which you work?	*n* = 257	Percent

Solo	37	16%
2–4 GPs	91	38%
>4GPs	111	46%
No response	18	

Do you see mostly younger, mostly older, or a mixture of patients in your practice?	*n* = 257	Percent

Mostly young (<25)	10	4%
Mostly older (>65)	45	19%
Mix of ages	184	77%
No response	18	

**Table 2:** GPs' attitudes towards their role of assessing fitness to drive and subsequent consequences (*n* = 217).

Question	Strongly agree/agree		Disagree/strongly disagree		No response	
I am confident in my ability to evaluate the driving fitness of my patients	114	53%	97	45%	6	2%
I know about alternative transport options in the local area for patients who can no longer drive	171	79%	44	20%	2	1%
I am concerned about my legal liability in relation to assessing fitness to drive	160	74%	55	25%	2	1%
A GP should be the initial person who assesses fitness to drive	135	62%	80	37%	2	1%
GPs should be primarily responsible for deciding who is fit to drive	45	20%	169	79%	3	1%
OTs should be primary responsible for deciding who is fit to drive	121	56%	90	41%	6	3%
It is the patient's responsibility to report medical conditions to VicRoads	185	85%	28	13%	4	2%
Medical practitioners who have been specially trained for assessing fitness to drive should be primarily responsible for making this decision	144	67%	70	32%	3	1%
Reporting a patient who I consider an unsafe driver to the driver licensing authority negatively impacts on the doctor-patient relationship	170	79%	45	20%	2	1%
I have been unduly pressured by patients to reconsider my decision to report them	99	46%	112	52%	6	2%
Revoking a patient's licence often leads to negative consequences for the patient	171	79%	44	20%	2	1%
I have had patients leave my practice over revoking their licences	50	23%	161	75%	6	2%
There are implications for vehicle insurance if a patient is unfit to drive	202	94%	9	4%	6	2%
I contact the Driver Licensing Authority in Victoria to seek guidance or advice	113	52%	98	46%	6	2%
